# A case report of intersigmoid hernia treated using laparoscopic surgery

**DOI:** 10.1016/j.ijscr.2021.105822

**Published:** 2021-03-26

**Authors:** Hiroyuki Kambe, Koji Kitamura, Satoshi Kaihara

**Affiliations:** Department of Surgery, Kobe City Medical Center General Hospital, Japan

**Keywords:** Intersigmoid hernia, Internal hernia, Computed tomography, Laparoscopy, Ischemia

## Abstract

**Introduction and importance:**

Intersigmoid hernia (ISH) is a rare disease that is difficult to diagnose preoperatively and sometimes causes intestinal necrosis that requires emergency surgery.

**Case presentation:**

The patient was an 87-year-old male with no history of abdominal surgery who visited our emergency outpatient service due to left lower quadrant pain and vomiting as chief complaints. Abdominal findings showed tenderness with the severest point in the left lower quadrant of the abdomen. Contrast-enhanced CT showed poor imaging of the dorsal sigmoid colon and an expanded proximal small intestine, with regional ascites around the small intestines. The patient was diagnosed with small bowel obstruction associated with ISH incarceration and underwent emergency surgery. Invagination of the small intestine into the intersigmoid fossa was found by laparoscopy. The incarcerated part was removed and the hernia orifice was sutured and closed. Mild congestion was seen in the incarcerated small intestine, but with no findings of ischemia. Thus, intestinal resection was determined to be unnecessary. The postoperative course was good and the patient was discharged on postoperative day 6.

**Clinical discussion:**

ISH is often diagnosed as simple ileus at the initial visit, which can result in delayed surgery. There are no case reports of complete remission of ISH with conservative therapy, and treatment with surgery is generally required. Our patient underwent early surgery because of CT findings that were characteristic of ISH and allowed diagnosis before surgery.

**Conclusion:**

Early diagnosis of ISH and performance of laparoscopic surgery can avoid the need for intestinal resection.

## Highlights

• Intersigmoid hernia (ISH) is difficult to diagnose preoperatively.
• An 87-year-old male was diagnosed with ISH based on CT findings.
• Invagination of the intestine in the sigmoid mesentery was found by laparoscopy.
• There is no case report indicating improvement of ISH by conservative therapy.
• Early surgery for ISH may reduce the need for intestinal resection.

## 1 Introduction

Internal hernia (IH) is a cause of acute abdominal symptoms and a rare disease that accounts for 0.5–5.8% of cases of small bowel obstruction (SBO) [1]. Strangulated ileus caused by IH may result in intestinal necrosis and requires early diagnosis. Intersigmoid hernia (ISH) is a internal hernia arising in the sigmoid mesentery and is often difficult to diagnose preoperatively because it is a rare disease and imaging diagnosis is difficult. Here, we report a patient with ISH incarceration who underwent emergency surgery after an early diagnosis. The work is reported in line with the the SCARE criteria [2].

## 2 Presentation of case

**Fig. 1:**
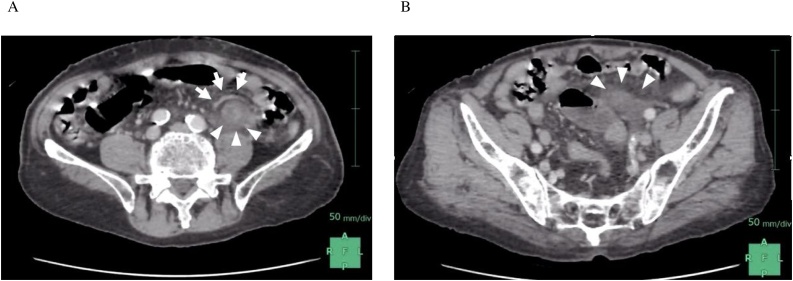
Contrast-enhanced CT on arrival. A. The herniated small intestine (arrows) on the dorsum of the vessel (arrowheads) in the sigmoid mesentery. B. Regional ascites around poor CT imaging of the herniated small intestine (arrows).

The patient was an 87-year-old male with no history of abdominal surgery who visited our emergency outpatient service due to left lower quadrant pain and vomiting as chief complaints. He was taking an anti-hypertensive drug. There was no family history of pancreatic cancer or genetic disorders. Abdominal findings showed tenderness that was most severe in the left lower quadrant of the abdomen, but no rebound tenderness. Blood tests showed CRP of 0.03 g/L and WBC 5100/μl, Lac of 2.0. A CT scan was performed, considering ileus, internal hernia, and intestinal perforation as differential diagnosis.　Contrast-enhanced CT revealed poor imaging of the dorsal sigmoid colon, an expanded proximal small intestine, and regional ascites around the small intestines (Fig. 1). Based on these findings, the patient was diagnosed with SBO associated with ISH incarceration and underwent emergency surgery 4 h after arrival at hospital and 13 h after onset. We obtained informed consent from the patient before surgery.

**Fig. 2:**
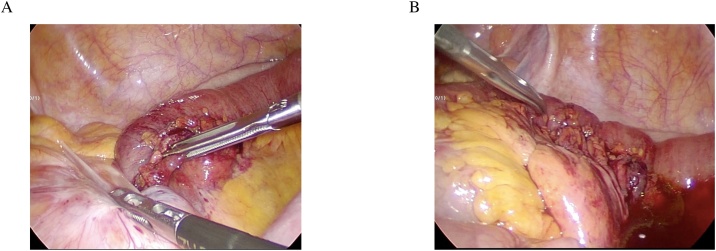
A: It was difficult to drag the small intestine directly from the orifice. B: There was no indication of intestinal necrosis.

A 12-mm port was inserted into the umbilicus and two 5-mm ports were inserted into the right abdomen. Findings in the peritoneal cavity included a partially expanded small intestine and small amounts of serous ascites. A part of the sigmoid mesentery bulged ventrally and formed a convex shape. On the dorsal side of the sigmoid-descending junction, a space occurred at a physiologic adhesion of the sigmoid colon and retroperitoneum, which formed the hernia orifice that caused an incarcerated small intestine of 10 cm in width (Fig. 2). It was difficult to drag the small intestine directly from the orifice; consequently, the incarceration was removed by incising the outer space of the sigmoid colon and expanding the orifice. Congestion was found in the incarcerated small intestine, but there was no indication of intestinal necrosis; therefore, intestinal resection was not performed. The hernia orifice was sutured and the surgery was completed. The operative time was 1 h 21 min and the hemorrhage volume was small. The surgery was performed by the senior surgical resident and Board Certified Surgeon in Gastroenterology.

The patient had a good outcome after surgery. The patient was elderly, and postoperative disuse was feared, but pain control was good and rehabilitation was possible with the use of intravenous acetaminophen 1000 mg four times a day for several days after surgery. There was no ischemia in the intestine where the strangulation was released, no postoperative ileus, he began to eat on postoperative day (POD) 3 and was discharged on POD 6. After discharge, the patient was followed up one month later, and outpatient follow-up was completed with no findings of ileus based on symptoms and abdominal findings. At 18 months postoperatively, a CT scan was performed by another department, which showed no recurrence of sigmoid mesenteric hernia at that time.

## 3 Discussion

**Fig. 3:**
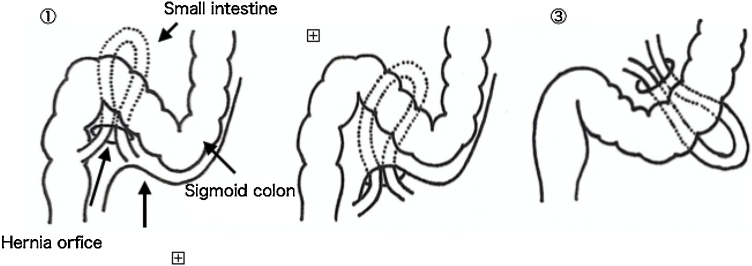
Classification of IH arising in the sigmoid mesentery. (1) Intersigmoid Hernia (2) Intramesosigmoid Hernia (3) Transmesosigmoid Hernia.

Benson and Killen [3] classified IH arising in the sigmoid mesentery into three categories: ISH, in which the intestine invaginates into a congenital fossa, the intersigmoid fossa, situated at the attachment of the lateral aspect of the sigmoid mesocolon; intramesosigmoid hernia, in which the intestine invaginates into a hole penetrating the left or right lobe of the sigmoid mesentery; and transmesosigmoid hernia, in which the intestine invaginates into a hole penetrating both the left and right lobes of the sigmoid mesentery (Fig. 3). These classes account for 6% of all internal hernias [4]. The hernia in our patient was ISH because the hernia orifice was located on the fusion fascia in the dorsal sigmoid colon where the small intestine was incarcerated.

A fossa in the sigmoid mesentery is thought to be formed through fusion of the left peritoneal surface of the sigmoid mesentery with the parietal peritoneum of the postural abdominal wall. The incidence is relatively high, ranging from 50% to 75% of all cadavers [5,6]. CT has an especially important role for diagnosis of acute abdominal symptoms, but there is difficulty with diagnosis of IH. Okada reported a preoperative diagnostic rate of IH related to the sigmoid mesentery of 15.9% [7]. Abdominal CT findings characteristic of ISH include formation of an edematous small bowel mass associated with wall thickness in the anterior side of the psoas major muscle; anteroinferior displacement of the sigmoid colon; convergence toward the medial side of the mesentery and mesenteric blood vessels; and caliber changes in the proximal and distal small intestine [8]. Our case was diagnosed as ISH due to poor CT imaging of the small intestine on the dorsum of the sigmoid mesentery and the left anterior side of the psoas major muscle; an expanded proximal small intestine; and regional ascites around the small intestine with poor CT imaging.

A search for “intersigmoid hernia” in PubMed and Japana Centra Revuo Medicina (original articles only) identified 39 articles from 1989 to 2020 (Table 1); therefore, 40 cases were evaluated after adding the current case. The median age was 51 years old (26–87) and the male-to-female ratio was 26:14. At the initial visit, 13 patients were diagnosed with ISH or hernia related to the sigmoid mesentery, and 20 patients were diagnosed with ileus or SBO. After admission, 13 patients underwent emergency surgery without conservative therapy, including 8 diagnosed preoperatively with ISH or hernia related to the sigmoid mesentery, and 5 diagnosed with strangulated ileus or IH. The other 27 patients received conservative therapy, but none were improved by this therapy and all were treated surgically after a median period of 6 days (0–27 days, excluding 4 cases without data). Of the 27 original conservative therapy cases, 22 underwent preoperative intestinal decompression. Seven patients required intestinal resection during surgery, and 6 of these cases were operated on after conservative therapy. Of all 40 patients, 22 underwent laparoscopic surgery, including all 13 patients treated after 2016.

There is no case report indicating improvement of ISH by conservative therapy. The period until surgery was relatively long for patients with ISH, and most patients who required intestinal resection were operated on after conservative therapy. In our patient, early preoperative diagnosis due to findings characteristic of ISH on CT may have permitted avoidance of intestinal resection. Therefore, it is important to consider the possibility of ISH in a case without a history of abdominal surgery. With regard to operative procedures, all patients underwent laparoscopic surgery regardless of preoperative intestinal decompression since 2016. This indicates that laparoscopic surgery can be applied to all patients without perforation if the working space can be secured.

## 4 Conclusion

Early diagnosis and laparoscopic surgery for a patient with ISH led to avoidance of intestinal resection. The possibility of ISH should be considered in a patient diagnosed with ileus who has no history of laparotomy.

## Declaration of Competing Interest

The authors have no conflict of interest regarding the work described in the manuscript.

## Sources of funding

This research did not receive any specific grant from funding agencies in the public, commercial, or not-for-profit sectors. Funding was from institutional sources only. The funding source had no influence on the study design; collection, analysis and interpretation of data; writing of the report; and decision to submit the article for publication.

## Ethical approval

This study has already got the ethical approval from the ethics committee of Kobe City Medical Center General Hospital.

## Consent

Written informed consent was obtained from the patient for publication of this case report and accompanying images. A copy of the written consent is available for review by the Editor-in-Chief of this journal on request.

## Author contribution

Hiroyuki Kambe and Koji Kitamura performed the surgery on this patient. Hiroyuki Kambe and Koji Kitamura and Satoshi Kaihara managed the postoperative course of the patient. Hiroyuki Kambe and Koji Kitamura wrote the manuscript. All authors have read and approved the final manuscript.

## Registration of research studies

Not applicable.

## Guarantor

Hiroyuki Kambe (Corresponding author and Guarantor).

## Provenance and peer review

Not commissioned, externally peer-reviewed.
